# Relationship between overall diet quality and composition and diarrhea in American adults: a cross-sectional study

**DOI:** 10.3389/fnut.2025.1570733

**Published:** 2025-05-23

**Authors:** Jincheng Wu, Xiaomin Lin, Qingfeng Zeng, Xianghui Zeng, Gang Cao, Muchu Xie, Jianping Luo, Lihua Li, Guiping Zeng, Shili Liu

**Affiliations:** ^1^Department of Cardiology, Ganzhou Hospital of Traditional Chinese Medicine, Ganzhou, Jiangxi, China; ^2^Department of Dermatology, Ganzhou Maternal and Child Health Care Hospital, Ganzhou, Jiangxi, China; ^3^Department of Anorectal Surgery, Ganzhou Hospital of Traditional Chinese Medicine, Ganzhou, Jiangxi, China; ^4^Department of Cardiology, Ganzhou People’s Hospital, Ganzhou, Jiangxi, China; ^5^Department of Radiology, Ganzhou Nankang Hospital of Traditional Chinese Medicine, Ganzhou, Jiangxi, China; ^6^Department of Pharmacy, Ganzhou Nankang Hospital of Traditional Chinese Medicine, Ganzhou, Jiangxi, China; ^7^Department of Cardiology, Hangzhou Mingzhou Naokang Recovery Hospital, Hangzhou, China

**Keywords:** diarrhea, Healthy Eating Index 2015, whole grains, refined grains, gut health

## Abstract

**Background:**

Diarrhea, as a significant aspect of digestive system disorders, the relationship between dietary quality and gut health has attracted increasing attention. This study aims to investigate the association between the Healthy Eating Index 2015 and its components and the risk of diarrhea.

**Methods:**

This study data from the 2007 to 2010 National Health and Nutrition Examination Survey databases, which included 7,395 adult participants. Diarrhea was defined according to the Bristol Stool Form Scale (BSFS) from the Gut Health Questionnaire. The HEI-2015 score was estimated based on two 24-h dietary recall interviews. surveylogistic regression models were employed to examine the association between the HEI-2015 and its components and the risk of diarrhea.

**Results:**

The results of this study indicated that, the HEI-2015 was significantly associated with a reduced risk of diarrhea. Specifically, a 1-point increase in the HEI-2015 score was associated with a 1% decrease in the odds of diarrhea (OR:0.99, 95% CI:0.98–0.99). A 1-point increase in the whole grain score was linked to a 5% reduction in the odds of diarrhea (OR:0.95, 95% CI:0.91–0.99), while a 1-point increase in the refined grain score was associated with a 3% reduction in the odds of diarrhea (OR:0.97, 95% CI:0.94–0.99).

**Conclusion:**

This study adds further evidence to the health benefits of whole grains and the potential risks associated with refined grains. As part of a balanced diet, promoting whole grain consumption could have a significant impact on public health by reducing the incidence of diarrhea and improving overall well-being.

## 1 Introduction

With ongoing socioeconomic development, an accelerated pace of life, increased mental stress, and unhealthy lifestyle factors, the prevalence of gastrointestinal diseases has been rising steadily (1–3). Among these, diarrhea, a significant component of digestive system disorders, has emerged as a major factor impacting individuals’ quality of life (4, 5). Currently, diarrhea is one of the most common health concerns globally (6). It not only causes frequent gastrointestinal discomfort but may also lead to malnutrition, physical exhaustion, and heightened psychological stress, which can severely affect both health and overall quality of life (7). Moreover, persistent or recurrent episodes of diarrhea may lead to dehydration, electrolyte imbalances, and nutritional deficiencies, all of which can compromise immune function and increase susceptibility to other illnesses (4, 8, 9). Furthermore, diarrhea places a considerable strain on healthcare resources, imposing an economic burden on both society and families (10).

Diarrhea is a common gastrointestinal disorder, typically characterized by increased stool water content, softened or watery stool consistency, and increased bowel movement frequency. Clinically, it is generally defined by one or more of the following criteria: increased stool frequency (≥3 bowel movements per day), altered stool consistency (soft, mushy, or watery), or increased stool weight (usually > 200 g/day) (11). While clinicians often rely on the standard of three or more loose stools per day, patients tend to associate diarrhea more with changes in stool consistency. In fact, stool consistency is primarily determined by its water-holding capacity, particularly the amount of non-bound or “free” water, which may represent the most physiologically accurate definition of diarrhea. However, quantifying this parameter in clinical practice is challenging; thus, the Bristol Stool Form Scale is recommended as a practical assessment tool (12). The etiology of diarrhea is highly complex, with various potential causes, including infections, dietary patterns, intestinal inflammation, and gut microbiota imbalance (13). Numerous modifiable factors related to the risk and management of diarrhea, such as dietary changes, pharmacological treatments, and physical therapies, have been extensively studied (8, 9, 14–16). As research on the mechanisms underlying diarrhea advances, the relationship between diet and gut health has garnered increasing attention (17–19). Recent research has emphasized the bidirectional relationship between diarrhea and the gut microbiota. Gut dysbiosis may precipitate diarrhea, while diarrhea in turn disrupts microbial balance. Additionally, dietary intake significantly influences microbial composition and activity, suggesting that nutritional status plays a central role in modulating diarrhea risk and severity (20–22). Thus, exploring the impact of dietary quality on diarrhea is of significant clinical and public health importance. In this study, we specifically evaluated the association between Healthy Eating Index 2015 component scores—including whole grains and refined grains—and the prevalence of diarrhea.

The Healthy Eating Index 2015 (HEI-2015) is an index developed by the U.S. Dietary Guidelines to assess dietary quality, consisting of 13 components (23). HEI has been shown to be associated with various diseases, including cardiovascular diseases, metabolic disorders, cancer, and anxiety/depression (24–27). However, the relationship between HEI-2015 scores and diarrhea remains underexplored. This study utilized data from the National Health and Nutrition Examination Survey (NHANES) and the Food Patterns Equivalent Database (FPED) to examine the association between overall dietary quality and the component groups measured by HEI-2015 with diarrhea in a nationally representative sample of U.S. adults.

## 2 Materials and methods

### 2.1 Data source

This is a cross-sectional study, and the data used in this research were obtained from the publicly available NHANES database. NHANES employs a complex, stratified, multistage probability cluster design to collect data for assessing the health and nutritional status of a nationally representative sample of the U.S. population. All NHANES protocols were approved by the Institutional Review Board of the National Center for Health Statistics, and participants provided written informed consent. The ethical approval of NHANES was granted by the US National Center for Health Statistics Research Ethics Review Board (Protocol No. 98-12, Protocol No. 2011-17).

**FIGURE 1:**
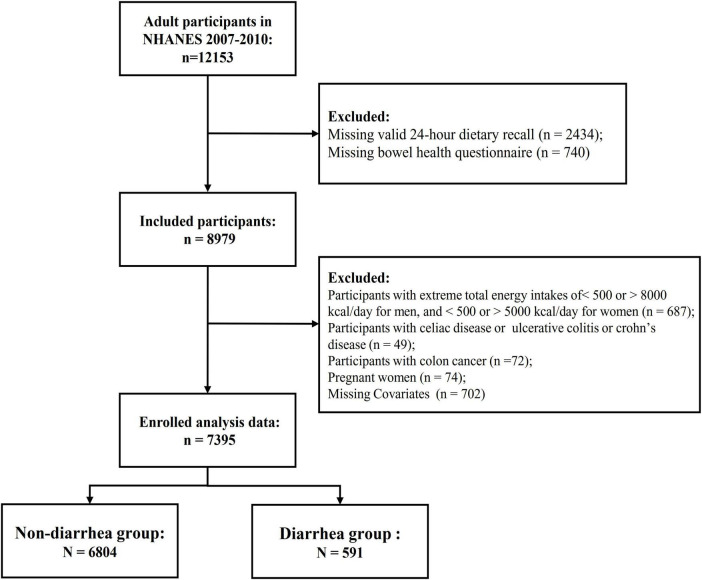
Study flowchart. NHANES, National Health, and Nutrition Examination Survey.

The data for this study were obtained from the 2007–2008 and 2009–2010 cycles, both of which included specific gut health questionnaires. Participants in the NHANES 2007–2008 and 2009–2010 database were included in this study if they completed the bowel health questionnaire and were 18 years of age or older. Participants with incomplete data on the gut health questionnaire, dietary recall, or physical activity questionnaire were excluded. Additionally, individuals who self-reported a history of inflammatory bowel disease, celiac disease, or colon cancer, or who had implausible energy intake levels (men and women with <800 or >4000 kcal, or <500 or >3500 kcal, respectively), or who were pregnant, were also excluded. In total, 7,395 participants were included in the analysis (Figure 1).

### 2.2 Definition of diarrhea

**FIGURE 2:**
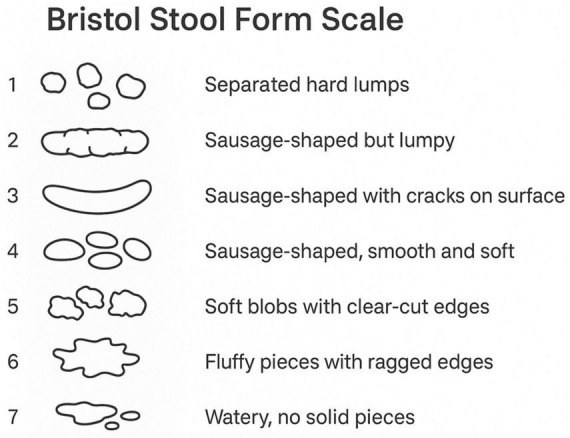
Bristol Stool Form Scale.

Participants were classified as having diarrhea based on their responses to the gut health questionnaire. The questionnaire was administered in the Mobile Examination Center (MEC) interview room using a computer-assisted personal interviewing (CAPI) system. Participants were shown a card with color images depicting the seven Bristol Stool Form Scale (BSFS) types and were asked (Figure 2), “Please look at this card and tell me the number that corresponds to your usual or most common stool type.” Diarrhea was defined as a usual or most common stool type of BSFS type 6 (fluffy pieces with ragged edges, mushy stool) or type 7 (watery, no solid pieces).

### 2.3 Healthy Eating Index-2015

Dietary intake data were obtained from two 24-h dietary recall interviews conducted by professional dietary interviewers as part of NHANES. The first interview was conducted in person, and the second interview was conducted by phone 3 to 10 days later, during which participants were asked to recall the types and quantities of foods and beverages consumed in the previous 24 h. The average of the two 24-h recall interviews was used to estimate dietary intake. Energy and nutrient intake for all foods were calculated using the Food and Nutrient Database for Dietary Studies (FNDDS). NHANES individual food data and the Food Patterns Equivalent Database (FPED) were then used to construct the intake of food components for the HEI-2015. The HEI-2015 assesses dietary quality and consists of 13 components, which are categorized into two types: adequacy components (total fruits, whole fruits, vegetables, total vegetables and legumes, whole grains, dairy, total protein foods, seafood and plant proteins, fatty acids) and moderation components (sodium, refined grains, saturated fat, and added sugars). Each component is assigned a different maximum score and weight. The total score, based on the 13 component scores (HEI-2015 score), ranges from 0 to 100, with higher HEI scores indicating better dietary quality (23).

### 2.4 Covariates

Demographic characteristics included in the analysis are age, sex (man or woman), race (Mexican American, Non-Hispanic White, Non-Hispanic Black, other races), education level (<12th grade, high school or above), and household income (<$20,000 or ≥$20,000). Lifestyle characteristics include smoking status, alcohol consumption status, and activity intensity. Smoking status is categorized as current smoker, former smoker, or never smoked. Alcohol consumption status is classified as current drinker, former drinker, or never drank. Body mass index (BMI) was calculated by dividing weight (kg) by height (m) squared. A BMI ≥ 30 was categorized as obesity, 30 > BMI ≥ 25 as overweight, 25 > BMI ≥ 18.5 as healthy weight, and BMI < 18.5 as underweight (28). Activity intensity was assessed using the Global Physical Activity Questionnaire (GPAQ) (29). Physical activity (PA) was calculated based on the MET value for each activity type, its weekly frequency, and duration using the following formula: PA (MET-min/week) = MET × weekly frequency of each PA × duration (30). The presence of stroke, chronic kidney disease, cardiovascular diseases, chronic obstructive pulmonary disease, diabetes, hypertension, hyperlipidemia, and depression was determined using a medical condition questionnaire, in which a doctor or healthcare professional asks: “Has a doctor or healthcare professional ever told you that you have any of these conditions?” Respondents who answered “Yes” were considered to have the condition. all original data collection instruments and protocols are available on the NHANES website.^1^

### 2.5 Statistical analysis

In this study, continuous variables are expressed as means with standard errors, while categorical variables are presented as counts with percentages. The baseline characteristics of the participants were described by categorizing them into diarrhea and non-diarrhea groups, with comparisons between groups conducted using non-parametric tests for continuous variables and chi-square tests for categorical variables.

Surveylogistic regression was employed to analyze the association between the HEI-2015 and its components with chronic diarrhea. Three models were used to assess this association. Model 1 was unadjusted for any confounders, Model 2 was adjusted for age, sex, race, education level, household income, smoking status, and alcohol consumption, and Model 3 was further adjusted for stroke, chronic kidney disease, cardiovascular disease, chronic obstructive pulmonary disease, diabetes, hypertension, hyperlipidemia, depression, BMI, and activity intensity, in addition to the adjustments made in Model 2. Restrictive cubic splines were utilized to explore potential non-linear associations between the HEI-2015 and chronic diarrhea, with all covariates in Model 3 adjusted for in the restrictive cubic spline plot. Statistical significance was defined as *P* < 0.05 for all analyses, All statistical analyses were conducted using SAS 9.4 (version 9.4, SAS Institute) and R studio (version 4.2.2).

## 3 Results

### 3.1 Baseline characteristics

In this study, a total of 7,395 participants were included, with an average age of 47.54 years; 3,729 were male (49.8%) and 3,666 were female (50.2%). Table 1 summarizes these characteristics. Participants who were older, had lower education levels, better family economic status, smoked, or had a history of alcohol consumption were more likely to experience diarrhea. Participants with a history of stroke, chronic kidney disease, cardiovascular disease, diabetes, hypertension, hyperlipidemia, or depression also had a higher risk of diarrhea. As shown in Table 1, participants with diarrhea tended to have lower HEI-2015 scores.

**TABLE 1:** General characteristics of the participants in the National Health and Nutrition Examination Survey 2007–2010.

	No, (survey weighted, %)			
		**Diarrhea**		
**Characteristic**	**Total**	**No**	**Yes**	***P*-value**
Age (years), mean (95% CI)	47.54 (0.40)	47.33 (0.42)	50.54 (1.02)	0.01
**Sex**				0.22
Man	3729 (49.80)	3468 (50.03)	261 (46.71)	
Woman	3666 (50.20)	3336 (49.97)	330 (53.29)	
**Races/ethnicity**				0.3
Non-Hispanic White	3791 (71.53)	3527 (71.83)	264 (67.36)	
Non-Hispanic Black	1302 (10.09)	1198 (9.97)	104 (11.81)	
Mexican American	1259 (8.24)	1134 (8.11)	125 (10.11)	
Other race	1043 (10.13)	945 (10.09)	98 (10.72)	
**Educations levels**				<0.01
Less than high school	845 (5.78)	710 (5.24)	135 (13.30)	
More high school or than	6550 (94.22)	6094 (94.76)	456 (86.70)	
**Annual family income**				0.02
Over $20,000	1786 (17.57)	1585 (17.07)	201 (24.40)	
Under $20,000	5609 (82.43)	5219 (82.93)	390 (75.60)	
**Smoking status**				0.04
Never	3915 (54.29)	3634 (54.90)	281 (45.75)	
Former	1969 (25.61)	1796 (25.40)	173 (28.56)	
Now	1511 (20.11)	1374 (19.70)	137 (25.68)	
**Alcohol drinker**				0.01
Never	975 (10.68)	870 (10.51)	105 (12.94)	
Former	1513 (16.87)	1373 (16.35)	140 (23.99)	
Now	4907 (72.46)	4561 (73.14)	346 (63.06)	
**Baseline comorbidities**				
Stroke	321 (3.28)	285 (3.06)	36 (6.24)	0.01
COPD	541 (6.75)	485 (6.62)	56 (8.61)	0.23
CKD	1390 (14.15)	1229 (13.61)	161 (21.51)	<0.01
CVD	900 (8.78)	813 (8.55)	87 (11.97)	0.07
Diabetes	1022 (9.48)	907 (9.01)	115 (16.08)	<0.01
Hypertension	3211 (35.90)	2897 (35.42)	314 (42.45)	0.03
Hyperlipidemia	5583 (73.44)	5093 (73.06)	490(78.77)	0.1
Depression	667 (7.55)	569 (7.07)	98 (14.12)	<0.01
**BMI status**				<0.01
Under weight	99 (1.29)	88 (1.29)	11 (1.29)	
Healthy weight	1869 (28.06)	1765 (28.64)	104 (20.07)	
Overweight	2589 (34.21)	2386 (34.45)	203 (30.91)	
Obesity	2838 (36.44)	2565 (35.62)	273 (47.74)	
**HEI 2015 total score**	54.01 (0.43)	54.16 (0.43)	51.84 (0.92)	0.01
**Physical activity, MET-min/week**	3577.20 (107.16)	3608.20 (107.31)	3149.03 (397.04)	0.25

Percentages and means (95% CI) were estimated using US population weights. *P*-values were computed separately for each covariate and indicate statistically significant differences between step groups if *P* < 0.05. Race/ethnicity was determined using preferred terminology from the National Center for Health Statistics as Non-Hispanic White, Non-Hispanic Black, and Mexican American. Mexican American individuals were oversampled rather than broader groups of individuals from Latin America. Other includes Asian, other Hispanic, Alaskan native, and multiracial individuals. Numbers in the table were unweighted. N, number; CI, confidence interval; CKD, chronic kidney disease; COPD, chronic obstructive pulmonary disease; CVD, cerebrovascular disease; HEI, Healthy Eating Index; MET, metabolic equivalent; BMI, body mass index.

### 3.2 Relationship between HEI-2015 and diarrhea

To examine the association between the HEI-2015 score and its components with diarrhea, logistic regression analysis was conducted using three models, with the results presented in Table 2. In Model 1, adjusted for age, sex, and ethnicity, HEI-2015 was significantly associated with a reduced risk of diarrhea. For each 1-point increase in the score, the odds of diarrhea decreased by 2% (OR: 0.98, 95% CI: 0.97–0.99). A 1-point increase in the whole grain score was associated with a 7% reduction in the odds of diarrhea (OR: 0.93, 95% CI: 0.89–0.98), while a 1-point increase in the refined grain score was associated with a 4% decrease in the odds of diarrhea (OR: 0.96, 95% CI: 0.93–0.99).

**TABLE 2:** Effects of the Healthy Eating Index and its components on the relationship between diarrhea.

		Model 1		Model 2		Model 3	
	**HEI score**	**OR (95%CI)**	***P*-value**	**OR (95%CI)**	***P*-value**	**OR (95%CI)**	***P*-value**
	**Total score**	**0.98 (0.97, 0.99)**	**<0.01**	**0.99 (0.98, 0.99)**	**0.01**	**0.99 (0.98, 1.00)**	**0.048**
	**Components**						
**1**	Total veg	0.93 (0.84, 1.04)	0.19	0.96 (0.86, 1.07)	0.45	0.96 (0.87, 1.08)	0.50
**2**	Green and bean	0.94 (0.88, 1.01)	0.07	0.95 (0.89, 1.02)	0.14	0.96 (0.9, 1.02)	0.17
**3**	Total fruit	0.97 (0.91, 1.04)	0.38	1(0.93, 1.07)	0.89	1.01 (0.94, 1.08)	0.77
**4**	Whole fruit	0.92 (0.85, 0.99)	0.03	0.94 (0.87, 1.01)	0.09	0.95 (0.89, 1.02)	0.17
**5**	**Whole grain**	**0.93 (0.89, 0.98)**	**<0.01**	**0.95 (0.9, 0.99)**	**0.02**	**0.95 (0.91, 0.99)**	**0.04**
**6**	Total dairy	0.98 (0.93, 1.02)	0.30	0.98(0.94,1.03)	0.43	0.98(0.94,1.03)	0.43
**7**	Total protein	1 (0.83, 1.21)	0.99	1.03(0.86,1.24)	0.72	1.03 (0.86, 1.23)	0.76
**8**	Sea plant protein	0.97 (0.91, 1.04)	0.41	0.99(0.93,1.06)	0.80	1 (0.94, 1.08)	0.90
**9**	Fatty acid	1 (0.97, 1.03)	0.82	1(0.97,1.04)	0.80	1.01 (0.98, 1.04)	0.65
**10**	Sodium	0.97 (0.92, 1.01)	0.16	0.96(0.92,1.01)	0.09	0.97(0.92, 1.01)	0.15
**11**	**Refined grain**	**0.96 (0.93, 0.99)**	**0.01**	**0.97 (0.94, 0.99)**	**0.03**	**0.97 (0.94, 0.99)**	**0.04**
**12**	Saturated fat	1 (0.96, 1.04)	0.89	1 (0.97, 1.04)	0.84	1.01 (0.97, 1.05)	0.54
**13**	Add sugar	0.97 (0.93, 1.01)	0.11	0.99 (0.95, 1.02)	0.48	0.98 (0.95, 1.02)	0.40

Bold font: *P* < 0.05. Model 1 was adjusted for age, sex, race. Model 2 was adjusted for age, sex, race, and education levels, annual family income, smoking status, alcohol consumption. Model 3 was adjusted for the variables in model 2 plus stroke, CKD, CVD, COPD, diabetes, hypertension, hyperlipidemia, depression, BMI, physical activity. NHANES, National Health, and Nutrition Examination Survey; OR, odds ratio; CI, confidence interval; CKD, chronic kidney disease; COPD, chronic obstructive pulmonary disease; CVD, cerebrovascular disease; HEI, Healthy Eating Index; BMI, body mass index.

In Model 2, which was further adjusted for education, annual income, smoking, and alcohol consumption, each 1-point increase in the HEI-2015 score was associated with a 1% reduction in the odds of diarrhea (OR: 0.99, 95% CI: 0.98–0.99). A 1-point increase in the whole grain score was associated with a 5% decrease in the odds of diarrhea (OR: 0.95, 95% CI: 0.90–0.99), and a 1-point increase in the refined grain score was associated with a 3% reduction in the odds of diarrhea (OR: 0.97, 95% CI: 0.94–0.99).

In Model 3, which was fully adjusted for other covariates, HEI-2015 remained significantly associated with a reduced risk of diarrhea. For each 1-point increase in the score, the odds of diarrhea decreased by 1% (OR: 0.99, 95% CI: 0.98–0.99). A 1-point increase in the whole grain score was associated with a 5% reduction in the odds of diarrhea (OR: 0.95, 95% CI: 0.91–0.99), while a 1-point increase in the refined grain score was associated with a 3% decrease in the odds of diarrhea (OR: 0.97, 95% CI: 0.94–0.99). Other components of the HEI-2015 did not show a significant association with diarrhea in Model 3.

**FIGURE 3:**
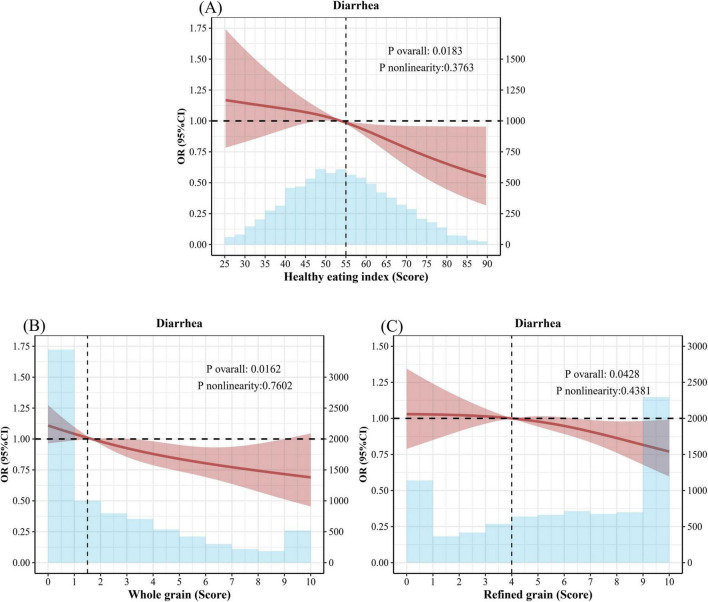
Healthy Eating Index and restrictive cubic splines for whole grains and refined grains and odds ratios (OR) for diarrhea. **(A)** Adjusted Spline Curves Analyze for the Association of Healthy Eating Index with diarrhea. **(B)** Adjusted Spline Curves Analyze for the Association of Whole grain with diarrhea. **(C)** Adjusted Spline Curves Analyze for the Association of Refined grain with diarrhea. Model was adjusted for age, sex, race, and education levels, annual family income, smoking status, alcohol consumption, stroke, CKD, CVD, COPD, diabetes, hypertension, hyperlipidemia, depression, BMI, physical activity. OR, odds ratio; CI, confidence interval; CKD, chronic kidney disease; COPD, chronic obstructive pulmonary disease; CVD, cerebrovascular disease; HEI, Healthy Eating Index; BMI, body mass index.

As illustrated in Figure 3, the restrictive cubic spline (RCS) plot revealed a linear relationship between the HEI-2015 score, whole grain score, refined grain score, and diarrhea. As the HEI-2015, whole grain score, and refined grain score increased, the risk of diarrhea decreased.

## 4 Discussion

Our study demonstrates a significant association between greater adherence to the HEI-2015 and a reduced risk of diarrhea. Specifically, the results underscore the important roles of whole grains and refined grains in this relationship. These findings align with existing literature, further supporting the health benefits of whole grains and the potential adverse effects of refined grains on gastrointestinal health (31–35).

Whole grains are rich in dietary fiber, vitamins, minerals, and phytochemicals, all of which significantly promote gastrointestinal health, particularly in areas such as digestion, modulation of gut microbiota, and reduction of colon cancer risk (32). Because whole grains retain the bran, germ, and endosperm, their dietary fiber content is higher (36). Dietary fiber can absorb water and form gels, thereby increasing the volume and moisture content of stool, helping relieve constipation and improve overall gut health. For some diarrhea patients, moderate dietary fiber may help absorb excess water, thereby alleviating diarrhea symptoms (37). In a systematic review and meta-analysis of prospective studies conducted by Dagfinn Aune et al. (38), 25 studies on dietary fiber or whole grain intake and colorectal cancer incidence were analyzed. The results showed that high intake of cereal fiber and whole grains is associated with a reduced risk of colorectal cancer (0.83–0.97, I^2^ = 0%). Furthermore, existing studies have shown that whole grain intake significantly reduces the risk of colorectal cancer (39, 40), and the occurrence of rectal cancer is often closely related to gut health.

Refined grains, including products such as white rice, white bread, and most commercial cereals, are processed to remove the bran and germ (36). This processing substantially reduces their fiber and nutrient content compared to whole grains. A high intake of refined grains has been linked to impaired gastrointestinal health and an elevated risk of digestive diseases (41). Andrew Reynolds et al. analyzed data from nearly 135 million person-years across 185 prospective studies and 58 clinical trials (involving 4,635 adult participants) and found that, when comparing the highest and lowest consumers of dietary fiber, all-cause and cardiovascular-related mortality, coronary heart disease incidence, stroke incidence and mortality, type 2 diabetes, and colorectal cancer incidence were reduced by 15%–30% (42). Additionally, Refined grains generally have a higher glycemic index, which causes a rapid spike in blood sugar levels, disrupting normal digestion and potentially triggering diarrhea symptoms (43). Research indicates that replacing refined grains with whole grains significantly improves gastrointestinal health, enhances stool quality, and reduces the risk of digestive system diseases (44).

Compounds such as phenolic acids in whole grains can be fermented by gut microbiota to produce short-chain fatty acids, such as butyrate, which positively influence gut health (45, 46). Studies have demonstrated that short-chain fatty acids not only improve the gut environment but also regulate the composition and activity of the gut microbiota, thereby enhancing overall gut health (45, 47). Furthermore, whole grain consumption is strongly associated with a reduced risk of various chronic diseases, including cardiovascular disease, type 2 diabetes, and certain types of cancer (39, 48–53). These health benefits are attributed not only to the effects of dietary fiber but also to the synergistic actions of other bioactive compounds in whole grains (42, 54). A study by Barbara Laddomada et al. reported that phenolic acids found in durum wheat and whole wheat flour exhibit significant anti-inflammatory effects (55). Specifically, certain levels of phenolic acids can notably inhibit the secretion of the pro-inflammatory cytokine IL-8, thereby offering important benefits to gut health.

In our analysis, certain components of the HEI-2015-such as Total Vegetables, Greens and Beans, Total Fruit, Whole Fruit, Total Dairy, Total Protein Foods, Seafood and Plant Proteins, Fatty Acids, Sodium, Saturated Fat, and Added Sugars—were not significantly associated with the risk of diarrhea. This lack of association does not necessarily imply a complete absence of biological relevance. Rather, it may suggest that the effects of these components on gastrointestinal health are indirect, potentially moderated by other dietary factors, host genetics, or lifestyle characteristics such as physical activity and stress levels (56).

Previous studies have shown that certain animal-based proteins can increase bile acid secretion and colonic microbial metabolism, which may, in some individuals, promote gut irritation and inflammation (57). On the other hand, plant-based proteins often contain fiber and phytonutrients that support gut barrier function and microbial diversity, though their isolated impact on diarrhea remains unclear (58). Similarly, seafood is rich in omega-3 fatty acids, which have recognized anti-inflammatory effects and may support gut integrity, but evidence regarding their direct role in reducing diarrhea is still limited and inconsistent (59). In addition, total vegetables and greens and beans are rich in fiber, vitamins, and polyphenols that help maintain mucosal integrity and promote gut microbial diversity (60). Similarly, fruits, including whole fruits, are excellent sources of soluble fiber and antioxidants. While moderate fruit intake generally supports gut health, overconsumption—particularly of high-fructose fruits—can contribute to osmotic diarrhea due to poor fructose absorption (61). Dairy intake is another complex factor. In lactose-intolerant individuals, consumption of milk and dairy products can lead to diarrhea through osmotic and fermentative mechanisms. Conversely, yogurt and fermented dairy products may support gut health by providing probiotics (62).

Our findings suggest that dietary interventions designed to increase whole grain intake while reducing refined grain consumption could be an effective strategy for mitigating the risk of diarrhea. Increasing whole grain intake and reducing refined grain consumption not only promotes gastrointestinal health but also provides a range of additional health benefits. Therefore, the role of whole grains should be emphasized in dietary recommendations, and individuals should be encouraged to make dietary choices that support overall gut health.

## 5 Conclusion

In conclusion, this study provides additional evidence supporting the health benefits of whole grains and the potential risks associated with refined grains. Future research should focus on further elucidating the biological mechanisms underlying these associations and examine the long-term effects of dietary patterns on gastrointestinal health. As a component of a balanced diet, promoting whole grain consumption may have a significant impact on public health by reducing the incidence of diarrhea and improving overall health outcomes.

## Data Availability

Publicly available datasets were analyzed in this study. This data can be found here: https://wwwn.cdc.gov/nchs/nhanes/ResponseRates.aspx.

## Footnotes

1
https://wwwn.cdc.gov/nchs/nhanes/Default.aspx
